# Low-Load Resistance Training With Blood Flow Restriction Improves Clinical Outcomes in Musculoskeletal Rehabilitation: A Single-Blind Randomized Controlled Trial

**DOI:** 10.3389/fphys.2018.01269

**Published:** 2018-09-10

**Authors:** Peter Ladlow, Russell J. Coppack, Shreshth Dharm-Datta, Dean Conway, Edward Sellon, Stephen D. Patterson, Alexander N. Bennett

**Affiliations:** ^1^Academic Department of Military Rehabilitation, Defence Medical Rehabilitation Centre, Headley Court, Epsom, United Kingdom; ^2^Department for Health, University of Bath, Bath, United Kingdom; ^3^Imaging Department, Oxford University Hospitals, Oxford, United Kingdom; ^4^School of Sport, Health and Applied Science, St. Mary’s University, London, United Kingdom; ^5^Faculty of Medicine, National Heart and Lung Institute, Imperial College London, London, United Kingdom

**Keywords:** blood flow restriction, musculoskeletal rehabilitation, hypertrophy, strength, function, pain, clinical outcomes

## Abstract

**Background:** There is growing evidence to support the use of low-load blood flow restriction (LL-BFR) exercise in musculoskeletal rehabilitation.

**Purpose:** The purpose of this study was to evaluate the efficacy and feasibility of low-load blood flow restricted (LL-BFR) training versus conventional high mechanical load resistance training (RT) on the clinical outcomes of patient’s undergoing inpatient multidisciplinary team (MDT) rehabilitation.

**Study design:** A single-blind randomized controlled study.

**Methods:** Twenty-eight lower-limb injured adults completed a 3-week intensive MDT rehabilitation program. Participants were randomly allocated into a conventional RT (3-days/week) or twice-daily LL-BFR training group. Outcome measurements were taken at baseline and 3-weeks and included quadriceps and total thigh muscle cross-sectional area (CSA) and volume, muscle strength [five repetition maximum (RM) leg press and knee extension test, isometric hip extension], pain and physical function measures (Y-balance test, multistage locomotion test—MSLT).

**Results:** A two-way repeated measures analysis of variance revealed no significant differences between groups for any outcome measure post-intervention (*p* > 0.05). Both groups showed significant improvements in mean scores for muscle CSA/volume, 5-RM leg press, and 5-RM knee extension (*p* < 0.01) after treatment. LL-BFR group participants also demonstrated significant improvements in MSLT and Y-balance scores (*p* < 0.01). The Pain scores during training reduced significantly over time in the LL-BFR group (*p* = 0.024), with no adverse events reported during the study.

**Conclusion:** Comparable improvements in muscle strength and hypertrophy were shown in LL-BFR and conventional training groups following in-patient rehabilitation. The LL-BFR group also achieved significant improvements in functional capacity. LL-BFR training is a rehabilitation tool that has the potential to induce positive adaptations in the absence of high mechanical loads and therefore could be considered a treatment option for patients suffering significant functional deficits for whom conventional loaded RT is contraindicated.

**Trial Registration:** ISRCTN Reference: ISRCTN63585315, dated 25 April 2017.

## Introduction

Functional ability during rehabilitation is closely associated with improvements in strength training (Kristensen and Franklyn-Miller, 2012). Therefore, optimizing the potential for adaptations in muscle strength is an important consideration in the progression of any musculoskeletal (MSK) rehabilitation program. It is widely accepted within both the exercise science and rehabilitation medicine domains that to elicit significant gains in muscle strength and hypertrophy requires lifting loads ≥70% of an individual’s 1-repetition maximum (1-RM) for a given movement (American College of Sports Medicine, 2009; Garber et al., 2011). However, for patients undergoing MSK rehabilitation, heavy-load resistance training (RT) can be contraindicated (Slysz et al., 2016) due to pain, muscle weakness and functional limitations preventing the attainment of these recommended heavier-loads (Hoyt et al., 2015). Patients with MSK injuries are often requested by their therapist to reduce the training load, potentially limiting the desired neuromuscular response to treatment and delaying the attainment of rehabilitation goals.

Blood flow restriction (BFR) exercise at low-loads (20–40% 1-RM) has been shown to be a safe (Loenneke et al., 2011) and effective tool to enhance the morphology and strength response in human muscle tissue (Slysz et al., 2016). However, the precise mechanisms underpinning the beneficial effects of BFR on skeletal muscle are unclear (Scott et al., 2015). A recent review reveals superior increases in muscle strength from heavy-load RT compared to low-load training with BFR, but comparable changes in muscular hypertrophy (Lixandrao et al., 2017). Low-load RT to volitional fatigue with and without BFR has demonstrated improvements in lower-limb muscle hypertrophy and endurance (Fahs et al., 2015). However, low-load RT with BFR was able to facilitate these improvements in muscle function using a reduced exercise volume (Fahs et al., 2015). There is now growing evidence for the practical and beneficial use of low-load blood flow restriction (LL-BFR) training as a clinical MSK rehabilitation tool (Takarada et al., 2000; Segal N. et al., 2015; Segal N.A. et al., 2015; Bryk et al., 2016; Giles et al., 2017; Tennent et al., 2017).

The majority of injuries in military populations occur in the lower limb (Anderson et al., 2016). There is subsequently a considerable economic and operational cost to the UK Ministry of Defence associated with lower-limb MSK injury. The Centre for Lower-Limb Rehabilitation at the UK Defence Medical Rehabilitation Centre (DMRC), Headley Court routinely treats and manages a large variety of lower-limb MSK disorders through 3 weeks multidisciplinary team (MDT) inpatient admissions. These injuries include, but are not limited to, overuse injuries (e.g., exertional lower-limb pain, patellofemoral pain, tendinopathy, and early osteoarthritis), bone fractures, post-surgical injuries (e.g., soft-tissue and ligamentous reconstruction) and hip and groin pain.

The development and investigation of emerging techniques with the potential to reduce recovery time and improve clinical outcomes is essential. To our knowledge, no studies have investigated the use of BFR exercise in a clinical population undergoing inpatient rehabilitation. Prior to the integration of novel techniques into clinical practice it is important to test the efficacy and safety against existing conventional training and rehabilitation methods. Therefore, we aimed to compare the effects of LL-BFR training with conventional heavy-load RT on changes in muscle volume and cross-sectional area (CSA), muscle strength and functional capacity in adults undergoing MSK inpatient rehabilitation. We also assessed the feasibility and adverse events associated with implementing LL-BFR exercise in a busy MDT rehabilitation setting.

## Materials and Methods

A detailed description of the study protocol including outcome measurement techniques and inclusion and exclusion criteria are published elsewhere (Ladlow et al., 2017). A description of the generic treatment pathway can be found in the **Supplementary File**.

### Trial Design

This is a parallel group, two-arm, assessor-blinded randomized controlled trial (RCT) with a two (group), by two (time) repeated measures design. The RCT was registered with ISRCTN Registry, trial number 63585315 and data collection occurred from August 2016 to February 2017. Ethical approval was provided by the UK Ministry of Defence research ethics committee (reference protocol number: 442/MODREC/13). Participants provided written informed consent and were randomly allocated to a conventional high-load RT or LL-BFR training groups. Outcome measurements were assessed at baseline and 3-weeks.

**FIGURE 1:**
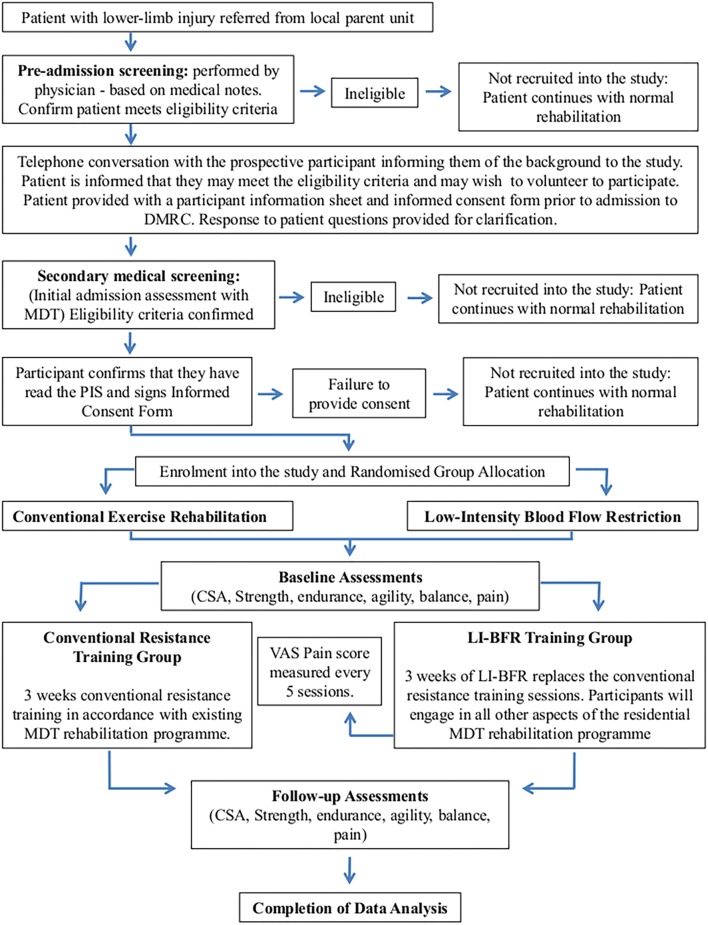
Study protocol and participant flow.

This study has been designed and reported in line with the CONSORT recommendations for reporting randomized trials (**Figure 1**).

### Participants

A heterogeneous group of 28 lower-limb injured male participants aged 19–49 years admitted for treatment at a MDT inpatient rehabilitation setting were recruited into the study. Typically these lower-limb injured participants present with a functional status enabling load-bearing RT but not at a level to allow a return to work. See **Table 1** for the study inclusion/exclusion criteria and **Table 2** for participant injury diagnosis.

**Table 1:** Inclusion and exclusion criteria.

**Inclusion criteria**
1. Male
2. Between 18 and 50 years of age
3. Serving regular UK Armed Forces personnel
4. Lower limb injury (patellofemoral pain, ACL reconstruction, ankle injury, projectile/blast related injury)
5. Referred to Defence Medical Rehabilitation Centre (DMRC), Headley Court for treatment
6. Engaged in a minimum of 4 weeks exercise rehabilitation at their local primary health care facility (PCRF) or regional rehabilitation centre (RRU)
7. Present with a level of function that would enable them to engage in load bearing conventional exercise rehabilitation
8. Unable to return to active duty due to persistent pain or muscular dysfunction
**Exclusion criteria**
1. Female
2. Prior history of cardiovascular disease (hypertension, peripheral vascular disease, thrombosis/embolism, ischaemic heart disease, myocardial infarction)
3. Have a personal history of the following musculoskeletal disorders: rheumatoid arthritis, avascular necrosis or osteonecrosis, severe osteoarthritis
4. Have a personal history of the following neurological disorders: peripheral neuropathy, Alzheimer’s disease, amyotrophic lateral sclerosis, multiple sclerosis, Parkinson’s disease, stroke, mild or severe traumatic brain injury
5. Have chronic or relapsing/remitting gastrointestinal disorders such as inflammatory bowel diseases, irritable bowel syndrome or gastrointestinal infections within 28 days of screening
6. Have an acute viral or bacterial upper or lower respiratory infection at screening
7. Have moderate or severe chronic obstructive pulmonary disease (COPD)
8. Amputation to the lower or upper extremity
9. ACL surgery within the last 4 weeks
10. Surgical insertion of metal components in lower limbs (may affect MRI results)
11. Have a personal history of any of the following conditions or disorders not previously listed: diabetes, fibromyalgia, active cancer, severe obesity (i.e., body mass index greater than 35 kg/m^2^), diagnosed mental illness (e.g., PTSD, depression, anxiety)
12. Have a current or previous use of any drugs known to influence muscle mass or performance within previous 6 months
13. Yet to receive any formal progressive exercise rehabilitation treatment within the past 4 weeks
14. Participants were excluded from the study if they were found to be at elevated risk of unexplained fainting or dizzy spells during physical activity/exercise that causes loss of balance


**Table 2:** Descriptive characteristics, injury diagnosis, muscle CSA/volume, strength measurements and functional performance status recorded at baseline.

Baseline measurements	LL-BFR	Conventional RT
**Participant characteristic (m ± *SD*)**
Participant numbers	14	14
Age (years)	33 ± 6	28 ± 7
Body height (cm)	178 ± 6	179 ± 7
Body mass (kg)	88 ± 19	92 ± 13
Body mass index (kg m^2^)	28 ± 5	29 ± 3
**Diagnosis, number (%)**
Exertional lower limb pain	6 (43)	6 (43)
Patellofemoral pain syndrome	3 (21)	1 (7)
Knee Surgery (e.g., ligament reconstruction)	2 (14)	3 (21)
Hip injury/surgery (e.g., arthroscopy)	2 (14)	3 (21)
Other lower-limb injury	1 (7)	1 (7)
Bilateral symptoms	9 (64)	8 (57)
**Baseline test performance scores (m ± *SD*)**
Quadriceps muscle CSA (cm^2^)^∗^	90 ± 17	95 ± 14
Quadriceps muscle volume (cm^3^)^∗^	2207 ± 486	2283 ± 400
Thigh muscle CSA (cm^2^)^∗^	200 ± 34	209 ± 27
Thigh muscle Volume (cm^3^)^∗^	5278 ± 1123	5330 ± 848
Leg-press 5-RM (kg)^∗^	78 ± 28	89 ± 33
Knee-extension 5-RM (kg)^∗^	27 ± 14	30 ± 11
Isometric hip extension (N)^∗^	265 ± 84	298 ± 87
Endurance (MSLT) (m)	1057 ± 461	1137 ± 600
Pooled Y-balance test (cm)^∗^	264 ± 29	280 ± 25


*^∗^Data reflects the injured limb only. LL-BFR, low-load blood flow restriction; RT, Resistance Training; CSA, cross-sectional area; RM, Repetition maximum; MSLT, multi-stage locomotion test.*

### Randomization, Blinding and Screening Process

Eligible participants were randomly allocated into either an LL-BFR or conventional training group using blocked randomization at a 1:1 ratio. Clinicians responsible for recording study outcome measures and magnetic resonance imaging (MRI) imaging were blinded to participant group allocation. All patients then engaged in additional activities associated with their MDT inpatient admission. The screening process involved the patient attending a physician led MD injury assessment clinical 4 weeks prior to admission for MDT inpatient treatment. The screening comprised of a standard assessment of the patients history, clinical assessments appropriate for the diagnosis, imaging and x-ray where available in accordance with the MOD best-practice care pathway for lower-limb MSK injury. If following this screening the patient met the eligibility criteria for entry into the study they were approached to provide written informed consent.

### Assessment Procedures

#### Determining Arterial Occlusion Pressure

The participant’s limb occlusion pressure (LOP) was determined prior to commencing the LL-BFR training program with the patient lying flat in a supine position. A 10-cm wide blood pressure cuff (Schuco TourniCuff, Schuco International, Watford, United Kingdom) was placed around the most proximal part of each thigh. The posterior tibial or dorsalis pedis pulse was located with a MD2 vascular doppler probe (Huntleigh Healthcare Ltd., Cardiff, United Kingdom). The tourniquet was rapidly inflated using a PTSii portable tourniquet system (Delfi Medical Innovations, Vancouver, BC, Canada) to a pressure of 250 mmHg (Noordin et al., 2009) such that the audible pulse was lost and then deflated until the pulse was regained; 60% of this LOP was calculated and used as the tourniquet pressure during the LL-BFR intervention (Scott et al., 2015).

#### Feasibility and Acceptability of LL-BFR Intervention

Acceptability was assessed by examining reasons for drop-out in any discontinuing participants and by comparing attrition rates between groups. Strengths, weaknesses and safety of the LL-BFR intervention was assessed by qualitative interviews with the project supervisor, lead exercise rehabilitation instructor, participant feedback and examination of adherence rates and adverse event reports.

#### Outcome Measures

All outcome measures were assessed at baseline and upon completion of 3-weeks inpatient rehabilitation using standardized and validated tests.

### Muscle Hypertrophy

#### Muscle CSA and Volume

For each slice of the injured limb, quadriceps and hamstring muscle compartments CSA (cm^2^) were measured and muscle compartment volumes calculated (cm^3^). Thigh CSA and volume encompassed both quadriceps and hamstring muscle architecture. Measurements were assessed prior to and 24-h following the completion of the 3-weeks rehabilitation program, using MRI with a GE Sigma scanner 1.5T (General Electric, WI, United States) in accordance with the method previously described by Abe et al. (2003). The same assessor completed baseline and post-intervention scans. All participants had both legs scanned with only the injured limb used for analysis purposes.

### Muscle Strength

#### 5-RM Knee Extension and Leg Press

Unilateral muscle strength was assessed using a dynamic 5-RM knee extension and a 45° incline leg press test (Pulse Fitness, Congleton, United Kingdom). An initial resistance was set based upon the result of a clinical assessment, pain intensity and participant feedback. The resistance was adjusted and test repeated until the participant was unable to complete five-repetitions. Participants received 3-min rest between each attempt and were allowed a maximum of three attempts to produce a 5-RM. This procedure followed established and widely used guidelines (Baechle and Earle, 2008).

#### Isometric Hip Extension

Unilateral isometric hip extension strength was assessed using a wireless digital microFET2 hand-held dynamometer (Hoggan Scientific LLC, Drapper, UT, United States) by the same assessor at baseline and post-intervention. The participant exerted a 5-s isometric maximal voluntary contraction (MVC) against the dynamometer and the examiner, whilst lying prone on a clinical examination couch as recommended by Thorborg et al. (2010). Participants performed four consecutive attempts with a 30-s recovery between attempts. Measures were reported as Newtons (N) with the highest value used for analysis.

#### Endurance

Endurance was measured using the multistage locomotion test (MSLT). The objective of this test was to assess the participant’s maximal walk/run distance (Vitale et al., 1997; Hassett et al., 2007). The test required the participant to walk/run on a 20-m track at gradually increasing speeds until they were unable to continue. Speed was controlled by paced-auditory cues accompanied by recorded verbal instructions. The test was terminated when the participant failed three consecutive attempts to reach the designated marker on the audible cue. Total distance covered in meters was recorded.

#### Balance

The Y-balance test assesses lower-body balance and flexibility using the Y-balance test kit (Plisky et al., 2009). Standing through a single supporting limb on the test kit, the participant reached with the free limb as far as possible along three lines positioned in anterior, postero-medial, and postero-lateral directions on each leg. To gain a global indicator of dynamic posture and balance, pooled data from all movement planes were calculated (distance performed in cm) and used for analysis.

#### Pain

A 100 mm horizontal visual analog scale (VAS) was used to measure pain and physical discomfort every five LL-BFR treatment sessions over the 3 weeks intervention (Collins et al., 1997). Using the VAS instrument, participants were asked “How do you rate the level of physical discomfort associated with the LL-BFR exercise,” immediately prior to starting the exercise, during the exercise and 5 min post-exercise. Symptomatic pain (the reproduction of pain at the associated site of injury) during LL-BFR was also assessed.

### Treatment Procedures

Before embarking on a fully powered RCT we wanted to assess the feasibility of BFR training against traditional RT methods employed in MSK rehabilitation. The primary aim of this RCT was to assess whether LL-BFR training is a rehabilitation tool that has the potential to induce positive adaptations in the absence of high mechanical loads (i.e., conventional RT). Therefore, we purposely selected a low-load non-weight bearing protocol in combination with BFR versus a traditional high mechanical load weight bearing protocol in our study to address the research question and properly assess the utility of BFR in our lower-limb injured patients. All patients recruited would have been functionally able to complete either intervention group. However, based on the exercises selected, the LL-BFR training protocol does not require upright mechanical loading whereas the conventional RT protocol does. To select two identical exercise protocols would not have addressed this fundamental question and is at the essence of this proof of concept RCT.

#### LL-BFR Training

A 10-cm wide contoured blood pressure cuff was placed around the proximal end of each thigh and inflated to the pre-determined 60% LOP. Participants performed low-load RT (30% 1-RM) combined with BFR using two exercises in sequence: (1) bilateral leg press using a leg press machine (Pulse Fitness, Congleton, United Kingdom), and (2) bilateral knee extensions using a knee extension machine (Pulse Fitness, Congleton, United Kingdom). 30% of 1-RM was determined based on an estimated 1-RM using their 5-RM muscle strength assessments. These exercises (one open chain quadriceps exercise and one closed chain with contributions from quadriceps and hip extensors muscles) enable RT to be performed with reduced axial loading. Off-loading an injured limb, whilst simultaneously provoking muscular overload is an essential component in the progression of many MSK rehabilitation programs. When full-loading bearing is not advised or contraindicated, these two exercises can be considered a suitable alternative (to traditional squatting, lunging, or deadlifts), and are frequently prescribed together in the prescription of lower-limb BFR training (Karabulut et al., 2010; Shimizu et al., 2016). Participants performed four sets of 30, 15, 15, and 15 repetitions at 30% of their predicted 1-RM (Segal N. et al., 2015; Segal N.A. et al., 2015; Giles et al., 2017; Tennent et al., 2017), with an inter-set interval of 30-s. A gradual progression of load lifted over the intervention period was permitted but based on patient feedback and clinician discretion. The inflation pressure was maintained for the duration of the exercise component and deflated during the 3-min inter-exercise rest interval.

The total length of time exposed to restricted blood flow was 4-min per exercise and 8-min per training session. Training was performed twice daily in the morning and afternoon (always separated by interludes of ≥5 h), from Monday to Thursday and once on Friday morning.

### Conventional (High-Load) RT

Participants completing conventional RT performed four-sets of three-exercises (deadlift, back squat, and lunges) three times per week. A gradual exercise progression using these closed chain exercises was determined by the exercise rehabilitation instructor based upon individual response to training. Repetitions per set were typically 6–8 and tailored to the individual needs of the patient with 3-min rest intervals between each set. The load lifted was a reflection of their best effort taking into account each individual’s injury limitations (for example, pain inhibition or inability to provide sufficient force due to weakness associated with their traumatized joint or muscle tissue). This protocol represents the type of exercise unavailable to patients suffering higher pain scores and lower levels of function.

Over the 15-days of supervised MDT rehabilitation participants completed a maximum of 23 8-min LL-BFR training sessions or 9 1-h conventional RT sessions. A full description of the standard 3-weeks MDT program, LL-BFR exercises, outcome measurement technique and example MRI images are provided in an online **Supplementary File**.

#### Sample Size

No formal sample size calculation determined by statistical assumptions and tests was performed as this was a pilot study design. Sample size recommendations for pilot RCTs were followed (Julious, 2005). Given the time constraints for data collection for this study we used a convenient sample with 14 participants recruited into each treatment group.

### Statistical Analysis

Results are presented using mean, SD and percentage change over time. Descriptive statistics were used to summarize eligibility, consent, randomization, adverse events, retention, completion, and intervention adherence rates. Participant demographic and baseline characteristics were also compared and reported. The results of strength, function and muscle volume/CSA tests were analyzed to evaluate group differences using a two-way repeated measures (time × group) analysis of variance (ANOVA). Even though there were no statistical differences between groups at baseline, analysis of covariance (ANCOVA) was used on muscle CSA, volume, strength and functional measurements to correct for any baseline differences and an adjusted post-intervention and change score reported as recommended by (Vickers and Altman, 2001). This statistical analysis of the data was exploratory only as our sample size did not allow for a definitive analysis. The level of significance was set at *p* < 0.05. All analysis was carried out using SPSS v.22.0.

## Results

### Baseline Data

**Table 2** summarizes the baseline demographic, diagnostic injury characteristics, muscle CSA/volume, muscle strength, and functional performance outcomes by group.

### Limb Occlusion Pressure

LL-BFR group participants had bi-lateral LOP measured (*n* = 28 limbs) before training commenced. After calculating 60% LOP, individualized tourniquet pressures ranged between 105 and 144 mmHg (mean: 124 ± 13 mmHg).

### Between Group Changes Over Time for All Outcomes

A two-way repeated measures ANOVA demonstrated no significant differences between groups for any outcome measure (*p* > 0.05). However, after adjusting for differences in baseline values there was a significant difference in mean quadriceps muscle volume [*F*(1,42) = 10.371,*p* = 0.002] after 3-weeks between LL-BFR and conventional RT group.

### Within Group Changes Over Time for All Outcomes

#### Muscle CSA and Volume

**FIGURE 2:**
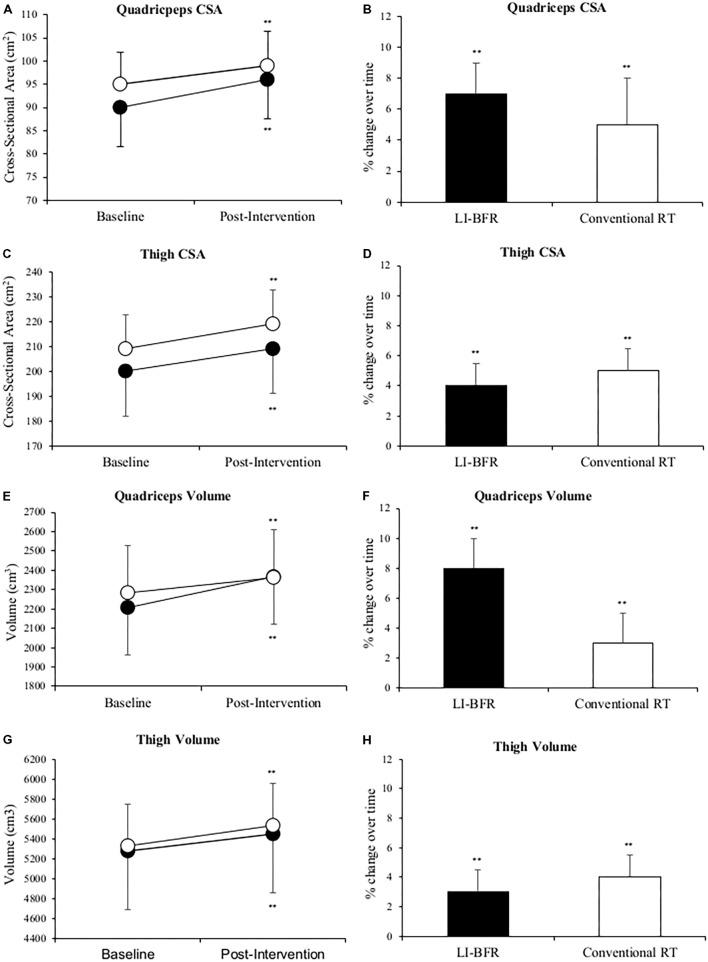
Changes in quadriceps muscle cross-sectional area (CSA) **(A,B)**, thigh CSA **(C,D)**, quadriceps muscle volume **(E,F)** and thigh muscle volume **(G,H)** at baseline and after 3-weeks of rehabilitation. Black points LL-BFR group, White points Conventional Resistance Training (RT) group. Bar charts show group percent changes over time. Data is expressed as mean ± SD. ^∗^*p* < 0.05 and ^∗∗^*p* < 0.01 versus baseline measurement. Data refers to primary injured limb. LL-BFR, low-load blood flow restriction; RT, resistance training; CSA, cross-sectional area.

A total of 45 injured limb (23 LL-BFR; 22 conventional RT group—some patients from each group presented with bilateral injuries) scores were analyzed. At 3-weeks both groups had significantly increased their quadriceps and thigh CSA and volume in the injured limb (*p* < 0.01). **Figure 2** shows quadriceps CSA increased 7 and 5%; quadriceps volume 8 and 3%; thigh CSA 4 and 5%; thigh volume 3 and 4% in the LL-BFR and conventional RT groups, respectively. After adjusting for baseline values, the adjusted change score between groups were comparable in CSA values, however, the LL-BFR group demonstrated a greater change score in quadriceps volume whilst the conventional RT group demonstrated a greater change score in thigh muscle volume (**Table 3**).

**Table 3:** Post-intervention values and change score of muscle cross-sectional area (CSA) and muscle volume after adjusted for differences in values scored at baseline (using analysis of covariance—ANCOVA).

	Adjusted pre-intervention value (m)	Intervention	Mean adjusted change score (95% CI)	Mean between group differences (95% CI)
Quadriceps muscle CSA (cm^2^)	92	LL-BFR	6 (4–8)	2 (-1 to 4)
		Conventional RT	4 (2–6)	
Quadriceps muscle volume (cm^3^)	2244	LL-BFR	160 (125–196)	82 (31–133)
		Conventional RT	79 (42–115)	
Thigh muscle CSA (cm^2^)	205	LL-BFR	9 (6–12)	1 (-3 to 5)
		Conventional RT	10 (7–13)	
Thigh muscle volume (cm^3^)	5303	LL-BFR	172 (109–234)	34 (-56 to 124)
		Conventional RT	206 (142–270)	


*Data reflects the injured limb only. Data presented as mean ± SE and 95% confidence intervals (CIs). LL-BFR, low-load blood flow restriction; RT, resistance training; CSA, cross-sectional area.*

#### Lower-Limb Muscle Strength

**FIGURE 3:**
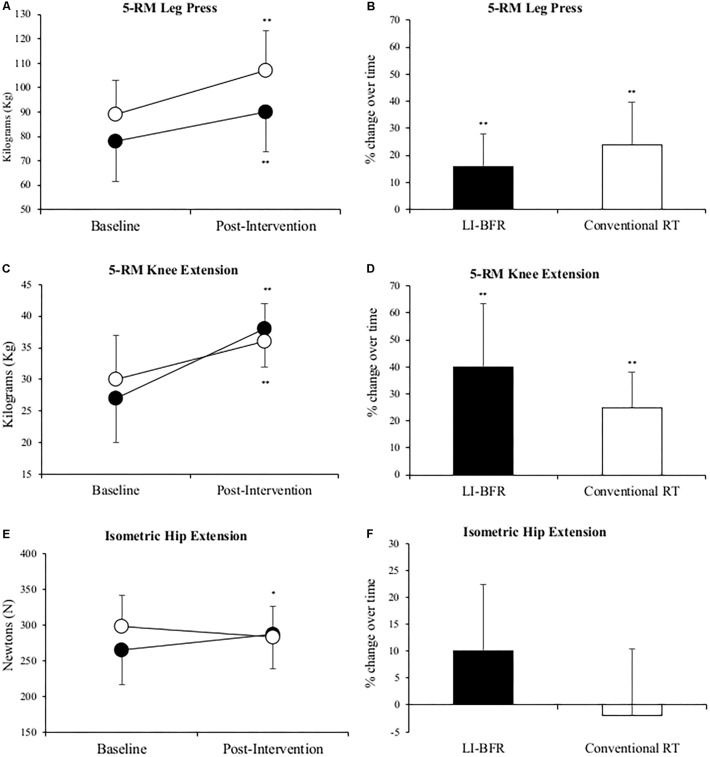
Changes in 5-RM leg press **(A,B)**, 5-RM knee extension **(C,D)** and isometric hip extension **(E,F)** at baseline and after 3-weeks of rehabilitation. Black points LL-BFR group, White points Conventional Resistance Training (RT) group. Bar charts show group percent changes over time. Data is expressed as mean ± SD. ^∗^*p* < 0.05 and ^∗∗^*p* < 0.01 versus baseline measurement. Data refers to primary injured limb. LL-BFR, low-load blood flow restriction; RT, resistance training; RM, repetition maximum.

**Figure 3** shows that mean 5-RM leg press and knee extension performance in the injured limb significantly improved in both groups (*p* < 0.01). Leg press strength improved 16 and 25%, knee extension strength improved 40 and 24% in the LL-BFR and conventional RT groups, respectively. Although positive changes in mean isometric hip extension strength (23 ± 66 N) were reported in the LL-BFR group but not in the conventional RT group (-17 ± 75 N), no significant changes occurred over time in either group (*p* > 0.05). After adjusting for baseline values, the conventional RT group demonstrated a greater mean change score in 5-RM leg press and the LL-BFR group demonstrated a greater mean change score in 5-RM knee extension (**Table 4**).

**Table 4:** Post-intervention values and change score of muscle strength and functional test after adjusting for differences in values scored at baseline (using analysis of covariance—ANCOVA).

	Adjusted pre-intervention value (m)	Intervention	Mean adjusted change score (95% CI)	Mean between group differences (95% CI)
Leg press 5-RM (kg)	88	LL-BFR	12 (6–19)	4 (-5 to 14)
		Conventional RT	16 (9–23)	
Knee extension 5-RM (kg)	29	LL-BFR	9 (6–12)	3 (-1 to 7)
		Conventional RT	6 (3–8)	
Isometric hip extension (N)	281	LL-BFR	18 (-11 to 47)	35 (-7 to 78)
		Conventional RT	-17 (-48 to 13)	
MSLT (m)	1085	LL-BFR	306 (140–472)	215 (-25 to 455)
		Conventional RT	91 (-81 to 264)	
Pooled Y-balance test s(cm)	272	LL-BFR	12 (1–22)	11 (-5 to 26)
		Conventional RT	1 (-9 to 12)	


*Data reflects the injured limb only. Data presented as mean ± SE and 95% confidence intervals (CIs). LL-BFR, low-load blood flow restriction; RT, resistance training; CSA, cross-sectional area; RM, repetition maximum; MSLT, multi-stage locomotion test.*

### Functional Outcomes

**FIGURE 4:**
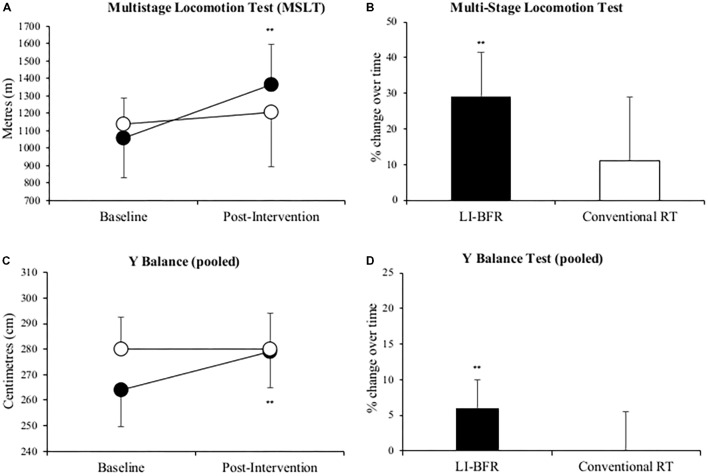
Changes in multi-stage locomotion test (MSLT) **(A,B)**, and pooled Y balance **(C,D)** at baseline and after 3-weeks of rehabilitation. Black points LL-BFR group, White points Conventional Resistance Training (RT) group. Bar charts show group percent changes over time. Data is expressed as mean ± SD. ^∗^ *p* < 0.05 and ^∗∗^*p* < 0.01 versus baseline measurement. Data refers to primary injured limb. LL-BFR, low-load blood flow restriction; RT, resistance training.

Mean MSLT distance significantly improved by 29% (306 ± 246 m, *p* = 0.01) in the LL-BFR group. The conventional RT group also recorded a greater mean distance covered after treatment (91 ± 341 m) but this change was non-significant (*p* > 0.05). LL-BFR group participants demonstrated a significant improvement (15 ± 20 cm, *p* = 0.03) in pooled Y-balance test scores, whereas conventional RT participant scores (-1 ± 32 cm, *p* = 0.93) did not improve (**Figure 4**).

#### Compliance/Acceptability/Feasibility and Pain Response to LL-BFR Exercise

**FIGURE 5:**
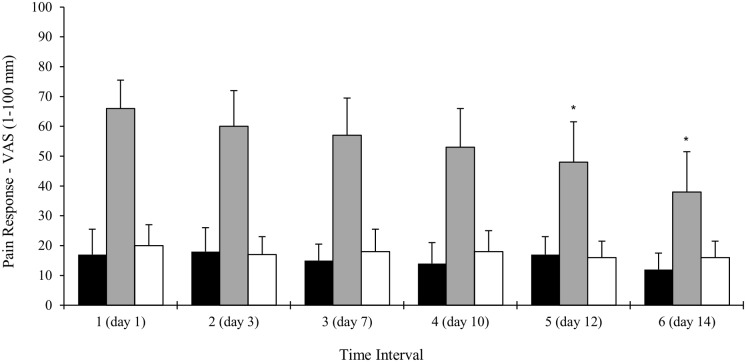
Changes in LL-BFR participant’s self-reported pain before, during and 5 min after the completion of exercise. Data collected over six time points (every five training sessions) during the 3-weeks intervention. Data is expressed as mean ± SD, ^∗^*p* < 0.05. VAS, visual analog scale.

Full patient compliance and adherence to the twice daily LL-BFR intervention was demonstrated. Mild muscular discomfort during exercise was reported (**Figure 5**) with self-reported pain returning to pre-exercise levels 5-min post-exercise. Mean symptomatic pain scores did not significantly change throughout the intervention (range: 13–19 mm). Pain reported during LL-BFR training was significantly greater (range: 44–66 mm) than pain reported before and after exercise (*p* < 0.01). When compared with baseline, there was a reduction in levels of muscular discomfort reported at commencement of the third week (day 10).

## Discussion

To our knowledge this is the first study using muscle volume and CSA, strength and functional capacity measures to demonstrate the application of LL-BFR when used as an adjunct to an inpatient musculoskeletal injury (MSKI) rehabilitation program. Both LL-BFR and conventional training groups showed significant within-group changes in muscle CSA/volume, 5-RM leg press and 5-RM knee extension scores after treatment. There were significant improvements in LL-BFR group participants MSLT and Y-balance test scores. The conventional training group functional capacity scores did not improve over time. Greater within-group changes and adjusted mean scores over time in the LL-BFR participants were observed; however, this does not constitute a superior training effect as the results of the two-way ANOVA showed no statistically significant differences between groups over time. Only after adjusting for baseline values was a significant difference between groups in quadriceps muscle volume found. Feasibility assessment revealed there were no drop-outs, no adverse events and full compliance associated with the LL-BFR intervention.

### Effects on Muscle Strength and Hypertrophy

Increasing muscle strength is a crucial aim of rehabilitation for all MSKI as muscle weakness is associated with delayed recovery and functional impairment (Kristensen and Franklyn-Miller, 2012). The comparable changes in clinical outcomes between LL-BFR and conventional RT in this study support previous findings in MSK rehabilitation research (Segal N. et al., 2015; Segal N.A. et al., 2015; Giles et al., 2017). Although greater mean change scores and percentage increases in 5-RM knee extension, isometric hip extension strength were not significantly different between groups they may be clinically relevant (Giles et al., 2017). Specifically, comparison of perceptual pain and perceived exertion responses during LL-BFR (30% 1-RM) versus heavy load (70% 1-RM) demonstrate these responses are generally lower in BFR groups compared with equivalent exercises at higher intensities (Hollander et al., 2010; Hughes et al., 2017; Giles et al., 2017). Therefore, the greater overall muscle strength gains in our BFR group may be due to lower joint forces and stress during BFR exercise allowing BFR participants to better tolerate these perceptual pain and exertion changes compared to the conventional training group. Whilst appealing, the current evidence-base supporting this position is limited (Hughes et al., 2017). However, Haun et al. (2017) found that muscle soreness reduced following post-exercise BFR, therefore BFR training could have a role in modulating pain.

The mean changes in quadriceps muscle CSA and volume (7 and 8%, respectively) in our study are comparable to those reported following a 12-days (twice daily) BFR-intervention in healthy subjects (Abe et al., 2005). A more recent RCT comparing conventional therapy with and without BFR after knee arthroscopy also reported greater hypertrophic gains (thigh girth at 6- and 16-cm proximal to patella pole) in a BFR treatment group (Tennent et al., 2017). Alongside our results, these findings demonstrate that short-term twice daily LL-BFR can result in significant hypertrophy adaptations in both healthy adults and lower-limb MSK injured patients. It is proposed that adaptations in muscle hypertrophy and strength are a result of metabolic stress associated with BFR and the mechanical tension of the load lifted acting synergistically to mediate numerous secondary mechanisms, all of which stimulate autocrine and/or paracrine actions to facilitate muscle growth (Pearson and Hussain, 2015).

Different mechanisms behind muscle hypertrophy and muscle strength in response to BFR training have been proposed (Jessee et al., 2018). The proposed mechanisms to elicit muscle hypertrophy include muscle cell swelling (Loenneke et al., 2012) and metabolite-induced fatigue (Abe et al., 2006). These mechanisms are considered to influence the intramuscular anabolic/anti-catabolic signaling response for protein synthesis (Fujita et al., 2007; Fry et al., 2010; Laurentino et al., 2012). The production of reactive oxygen species (ROS) (Kawada and Ishii, 2005; Pope et al., 2013) and post-exercise reductions in muscle oxidative stress and proteolysis (Haun et al., 2017) are also considered to influence muscle growth. The mechanisms behind strength adaptations and the role of the neuromuscular system are less clear. However, an acute bout of BFR training appears to increase corticomotor excitability (Brandner et al., 2015) and proposed to increase in fast twitch muscle fiber/motor unit recruitment (Takarada et al., 2002; Cook et al., 2013). However, a recent study by Hill et al. (2018) found no changes in muscle activation (EMG amplitude) or electrical efficiency during a 4 weeks BFR intervention and concluded that early phase increases in muscle strength are not associated with neural changes, but was likely a result of muscle hypertrophy. However, in the absence of research demonstrating a causal link, any suggested associations between BFR training and subsequent muscle growth are purely speculative.

### Effects on Physical Function Outcomes

In MSK rehabilitation practice much emphasis is placed on the importance of physical function. However, very few studies have assessed this component of treatment in BFR research. One study has demonstrated greater improvements in a timed stair ascent task following conventional therapy with BFR in knee OA patients (Tennent et al., 2017). In our study significant improvements in endurance (MSLT distance) were demonstrated in the LL-BFR group participants. It has been reported that favorable adaptations occur within vascular networks as a response to LL-BFR (Casey et al., 2010; Hunt et al., 2013). Although these mechanisms were not tested, it is possible this may have contributed toward improved endurance capacity changes in the LL-BFR group. We also found a non-significant improvement in mean isometric hip extension strength in the LL-BFR group (23 N). Previous research has reported strength and hypertrophic adaptations in the muscles located proximal to the applied pressure as a result of pre-fatiguing in the muscles below the cuff (Dankel et al., 2016). This enhanced stimulation of the hip musculature (located proximal to the cuff) may explain the significant improvements demonstrated in pooled Y-balance scores in the LL-BFR group relative to the conventional RT group. Enhanced hip muscle strength may also contribute to improvements in walking/running mechanics and therefore endurance capacity. Further research is required to better understand how muscle adaptations to BFR exercise influences functional capacity in MSK injured populations.

### Feasibility Components

Comparison of attrition rates between groups revealed no recorded drop-outs or any discontinuing participants from either group. Session adherence rates were 100% (LL-BFR) and >90% in the conventional training group. No adverse events or safety breaches were observed and LL-BFR participant mean pain scores during training reduced over time (**Figure 5**) suggesting a degree of adaptation to an unfamiliar exercise stimulus.

### Limitations

Our participants were suffering MSK injury of the lower-limb at the same stage of functional recovery, however, they comprised a mix of diagnostic injury types undergoing a complex multi-modal intervention and some heterogeneity in clinical severity may have attenuated the treatment effect. We did not follow-up our participants beyond the 3-weeks period of rehabilitation and no conclusions can be made on any longer-term benefits of treatment. Due to time-limited constraints for data collection we used a convenient sample. The small sample size limits the ability to make definitive statements regarding the effectiveness of LL-BFR and results may be susceptible to type I or type II errors. Whilst the use of a young male population may limit the generalizability to other populations and settings, we believe the findings are relevant to any MSK injured rehabilitation population. The use of different loading conditions and exercises between groups was intentional to properly address the aims of the study; however, we acknowledge a specificity of training effect may have led to the greater change scores for 5-RM knee extension and quadriceps muscle volume in the LL-BFR group. Future research investigating BFR training should consider the potential for a specific transfer of strength gains between training and testing. Also, due to insufficient data in the conventional RT group, comparisons in exercise volume were not possible; this should be a consideration in any future BFR related study using clinical populations.

## Conclusion

This is the first study to demonstrate that twice daily LL-BFR exercise at 30% 1-RM can be safely and effectively implemented into a busy inpatient MDT rehabilitation setting. Twice daily BFR training at low-load (30% 1-RM) resulted in significant improvements in lower-limb muscle hypertrophy, strength and function after 3-weeks inpatient rehabilitation. LL-BFR training yielded positive gains in participant physical function relative to conventional RT. Both conventional RT and LL-BFR can safely be used to improve clinical outcomes; however, LL-BFR training is a rehabilitation tool that has the potential to induce positive adaptations in the absence of high mechanical loads. This finding may have implications for patients suffering significant functional deficits for whom conventional training is contraindicated. Further studies using randomized designs examining the effects of LL-BFR training in patients with greater levels of impairment are needed.

## Author Contributions

PL, RC, SD-D, and SP conceived the study design. AB obtained the funding. SD-D and DC were responsible for recruiting and consenting participants into the study, and delivering the intervention. ES analyzed all MRI related outcomes. PL, RC, and ES analyzed the findings. PL and RC produced a draft manuscript. All authors read, critically reviewed, and approved the final version of the manuscript.

## Conflict of Interest Statement

The authors declare that the research was conducted in the absence of any commercial or financial relationships that could be construed as a potential conflict of interest.
