# Supplementary material for: Low-Load Resistance Training With Blood Flow Restriction Improves Clinical Outcomes in Musculoskeletal Rehabilitation: A Single-Blind Randomized Controlled Trial
Source: Front Physiol. 2018 Sep 10;9:1269. doi: 10.3389/fphys.2018.01269 (PMC6139300; doi:10.3389/fphys.2018.01269)
Supplement: Supplementary file 1 [file Presentation_1.PDF]

Supplementary File to:

LOW-LOAD RESISTANCE TRAINING WITH BLOOD FLOW RESTRICTION IMPROVES CLINICAL OUTCOMES IN MUSCULOSKELETAL REHABILITATION: A SINGLE-BLIND RANDOMIZED CONTROLLED TRIAL

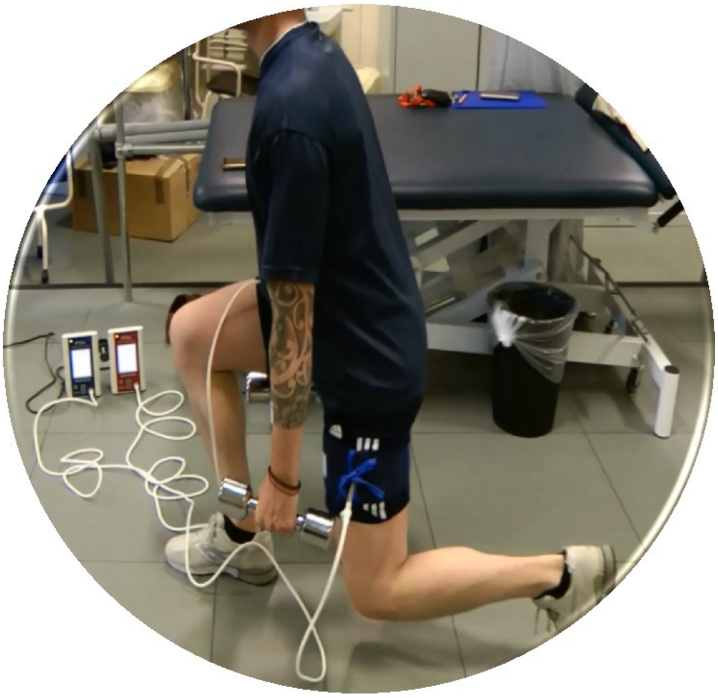

Supplementary File – Contents:

Annex A – Description of Generic DMRC Lower-Limb Rehabilitation …………………… page 3-4
MDT Programme (3-Weeks).

Annex B – LI-BFR Intervention Exercises…………………………………………………….page 5

Annex C – Y-balance test ……………………………………………………………......page 5

Annex D – Isometric Hip Extension with Hand-Held Dynamometry…………………………page 6

Annex E – Example MRI Output Image…………………………………………………….page 6

Annex A – Description of Generic DMRC Lower-Limb Rehabilitation MDT Programme (3-Weeks)

All participants will receive individualised programmes focussing on improving range of motion, balance, aerobic conditioning, manual therapy and education sessions. The generic MDT programme will be common to both groups with only the conventional RT or LI-BFR intervention individualised to each participant dependent on group allocation. Additional information regarding each of these components is provided below.

Stretching and range of motion exercise
Static and active stretching and foam-roller techniques will be employed to maintain the range of motion (ROM) required for optimal function. This forms part of routine clinical practice within lower-limb rehabilitation in the UK military.

Neuromuscular control and functional balance exercise.
Balance and proprioceptive exercises will be included to restore deficits and re-establish neuro-motor control. Progression will be applied by increasing the complexity and difficulty of the exercise, by reducing the base of support, adding dynamic movements on unstable surfaces, and increasing the range through which the movement is performed. Support for neuromuscular training in lower-limb rehabilitation has been reported in the literature [1].

Aerobic exercise
Participants will undertake light to moderate aerobic conditioning over the intervention period. In addition to the general health benefits conferred by aerobic exercise, moderate joint loading has been shown to be beneficial for joint health because of mechanosensitive chondroprotective pathways [2]. No study has described the optimal dose of aerobic exercise for patients undergoing lower-limb rehabilitation in terms of intensity, volume and duration. In this study, the supervising ERI will determine the nature of aerobic exercise (walking, cycling, swimming, cross-trainer), and progression in intensity based on individual examination finings and patient response to exercise.

Manual therapy
Manual therapy techniques will be used to modify the quality and range of motion of soft tissue structures, and assist with pain relief. The manual therapy intervention will be prescribed individually for each participant on the basis of the physical examination findings, from a list of techniques including, trigger point massage, passive joint mobilisation, distraction and sustained stretches [3]. These techniques are commonly used in the management of injured military personnel at DMRC and delivered by their respective MSK physiotherapist.

Education
Educating the patient on factors surrounding their treatment and the importance of regular exercise is a key component of the rehabilitation process at DMRC to optimise patient adherence to a home or work-based exercise programmes. Education and advice will be a focus of the intervention and will include information on diagnosis and aetiology of their injury, rationale for treatment, the benefits of exercise, joint protection and activity modification strategies, pain management, coping with acts of daily living (sitting, driving, sleeping, work), and the importance of increasing physical activity levels in everyday life [3]. Unsupervised home based prescription of BFR has not be investigated and therefore not recommended in the UK Defence best practice guidelines. Instead, the LI-BFR group will be prescribed a conventional resistance training programme to perform upon discharge from the rehabilitation centre.

Table 1 – Components of generic DMRC, MDT in-patient rehabilitation programme

**Treatment Modality**	**Treatment Content**	**Treatment Goals**	**Frequency per week (duration)**
Group Exercise: led by ERI	Group based circuit training that primarily involves high repetition muscular strengthening exercises targeting the whole body. May also include minor team games	The same as the Individual exercise sessions, but also the promotion of group cohesion and social support	12
Hydrotherapy / Swimming	Non-weight-bearing aerobic exercise, strengthening exercises, active range of motion exercises, self-paced recreational swimming, progressive/assisted weight-bearing exercise and activity	Improve muscle strength, improve aerobic capacity, increase joint range of motion, improve confidence in weight bearing, induce relaxation, and promote enjoyment and fun.	5
Individualised Physiotherapy	Manual therapy techniques, muscle activation and timing patterns, active and passive range of motion exercises, advice on home exercise, gait re-education training	Improve quality and timing of movement, improve muscle strength, reduce pain, increase joint range of motion, induce relaxation, promote normal walking gait.	5
Individualised Occupational Therapy Session	Relaxation techniques, postural re-education, cognitive–behavioural therapy techniques, self-help coping strategies, pain management	Induce relaxation, promote behavioural change, control pain, correct/improve poor posture	5
Patient Education	Coping with pain, benefits of exercise, joint protection, anatomy and pathology of hip pain, nutrition	Activity modification, reduction of pain, promote behavioural change, weight management, improve knowledge of treatment options, improve ability to relax, improve knowledge of self-help techniques	5

References:

1. Ageberg E, Nilsdotter A, Kosek E, Roos EM: Effects of neuromuscular training (NEMEX-TJR) on patient-reported outcomes and physical function in severe primary hip or knee osteoarthritis: a controlled before-and-after study. *BMC Musculoskelet Disord* 2013, 14:232.

2. Oiestad BE, Osteras N, Frobell R, Grotle M, Brogger H, Risberg MA: Efficacy of strength and aerobic exercise on patient-reported outcomes and structural changes in patients with knee osteoarthritis: study protocol for a randomized controlled trial. *BMC Musculoskelet Disord* 2013, 14:266.

3. Coppack RJ, Bilzon JL, Wills AK, McCurdie IM, Partridge L, Nicol AM, Bennett AN: A comparison of multidisciplinary team residential rehabilitation with conventional outpatient care for the treatment of non-arthritic intra-articular hip pain in UK Military personnel - a protocol for a randomised controlled trial. *BMC Musculoskelet Disord* 2016, 17:459.

Annex B – LI-BFR Intervention Exercises

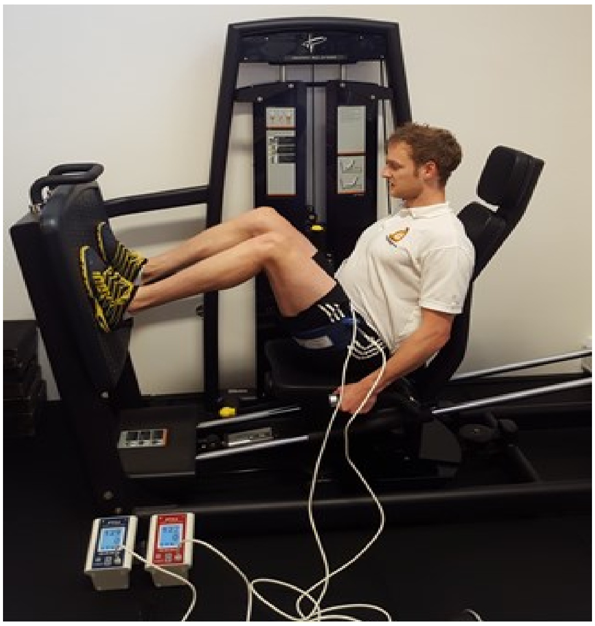

A. Seated Leg Press with BFR

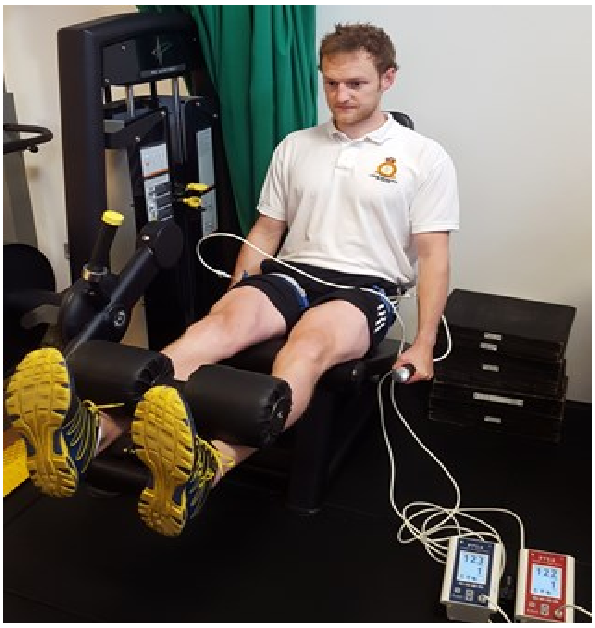

B. Seated Knee-Extension with BFR

Annex C – Y-balance test

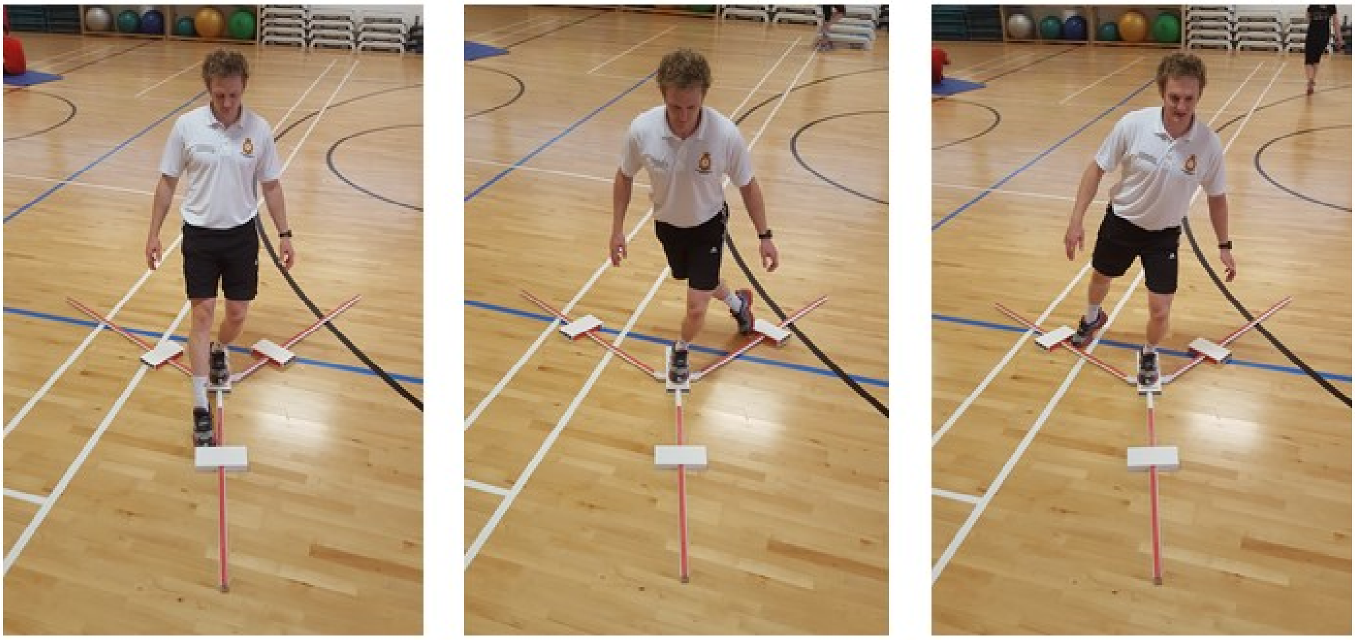

A B C

From a single-leg stance the participant reaches the freely movable limb along a line in the anterior (A), posterolateral (B), and posterolateral (C) directions.

Annex D – Isometric Hip Extension with Hand-Held Dynamometry

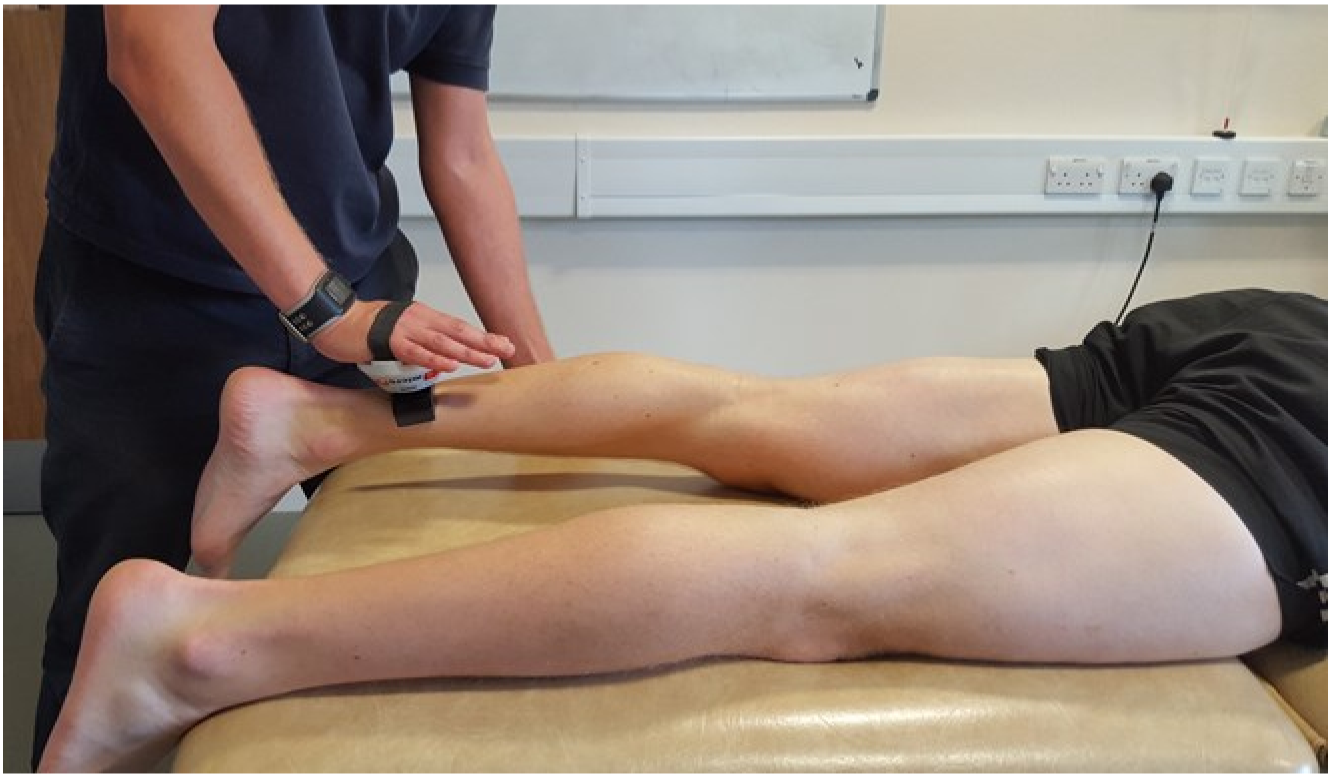

The examiner applies resistance in a fixed position whilst the participant exerts a 5 second isometric maximal voluntary contraction (MVC) against the dynamometer and the examiner. Participants will perform four consecutive attempts with a 30 second recovery between attempts. Strength measures will be reported as Newtons (N). The highest value will be used for analysis purposes.

Annex E – Example MRI Output Image

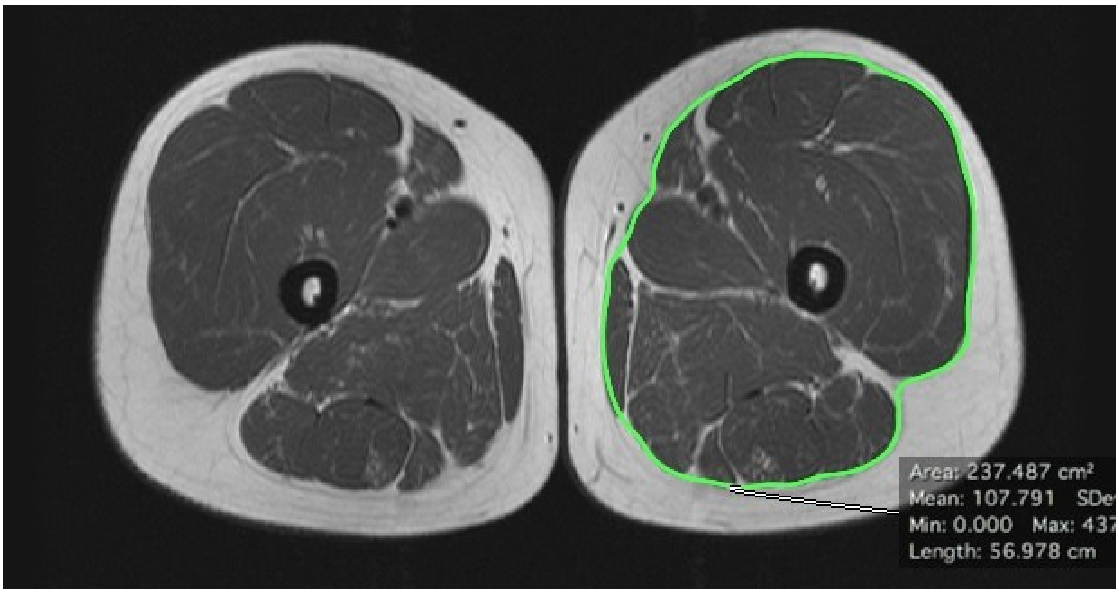

MRI image of total thigh cross-sectional area (CSA). The green line, drawn by a consultant radiologist, determines the CSA of the muscle.
